# Highlight: The Social Code—Deciphering the Genetic Basis of Hymenopteran Social Behavior

**DOI:** 10.1093/gbe/evac182

**Published:** 2023-01-10

**Authors:** Casey McGrath

*The authors of a new study set out to uncover early genetic changes on the path to sociality.*

Beginning with Darwin, biologists have long been fascinated by the evolution of sociality. In its most extreme form, eusocial species exhibit a division of labor in which certain individuals perform reproductive tasks such as egg laying, while others play nonreproductive roles such as foraging, nest building, and defense. This type of system requires individuals to forgo some or all of their own reproductive success to assist the reproduction of others in their group, a concept that at first glance seems incompatible with the key tenets of evolution (i.e., the drive of natural selection on individuals). Although the honeybee is perhaps the most well-known example of a social species, the honeybee's complex society represents just one end of a spectrum of social structures that can be observed among the Hymenoptera, which includes bees, wasps, and ants. At the other end are more rudimentary social structures involving, at the most basic level, the cooperation of just a few individuals and their offspring. Although most research to date on insect sociality has focused on more complex social systems, understanding the evolution of these more rudimentary forms will likely help to reveal the earliest changes on the path to sociality. The authors of a new study published in *Genome Biology and Evolution*, titled “Co-expression gene networks and machine-learning algorithms unveil a core genetic toolkit for reproductive division of labour in rudimentary insect societies,” set out to fill this gap (Favreau et al. 2023). According to first author Emeline Favreau, “Our work was unique in that we focused on six bee and wasp species that are not highly social, but have more rudimentary forms of cooperation, and are close relatives of highly social species.” By using machine learning algorithms to analyze gene expression across six species that represent multiple origins of sociality, the authors uncovered a shared genetic “toolkit” for sociality, which may form the basis for the evolution of more complex social structures.

The international team of researchers included Katherine S. Geist (co-first author) and Amy L. Toth from Iowa State University, Christopher D.R. Wyatt and Seirian Sumner from University College London, and Sandra M. Rehan from York University in Toronto. The authors worked together on this article “because we all find it important to understand the origins of sociality,” says Favreau. “We had been in the field observing the fantastic diversity of social lives, such as large nests of wasps busy with collective behavior or small carpenter bees organizing their broods in minute tree branches. We kept asking ourselves: But how did these behaviors come about? With this paper, we dove deep into the evolutionary stories to uncover molecular evidence of the emergence of social organization.”

**Fig. 1:**
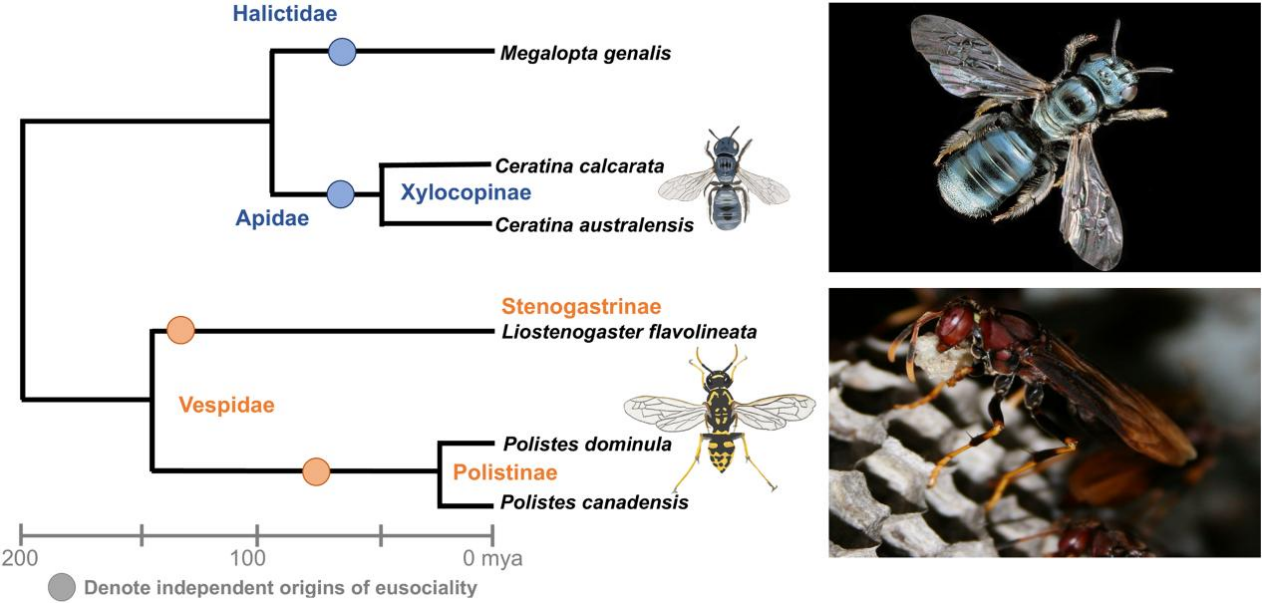
The study involved three bees and three wasps representing four independent origins of sociality (circles; nonsocial sister species not shown) and a range of social and ecological phenotypes (Drawings by Katherine S. Geist). Images shown are the bee species *Ceratina calcarata* (top; photo credit: Sandra Rehan) and the wasp species *Polistes dominula* (bottom; photo credit: Seirian Sumner).

The study involved a comparative meta-analysis of data from three bee species and three wasp species that represent four independent origins of sociality: the halictid bee *Megalopta genalis*, the xylocopine bees *Ceratina australensis* and *C. calcarata*, the stenogastrine wasp *Liostenogaster flavolineata*, and the polistine wasps *Polistes canadensis* and *P. dominula* (fig. 1). “Using data on global gene expression in the brains of different behavioral groups (reproducing and nonreproducing females), we found that there is a core set of common genes associated with these fundamental social divisions in both bees and wasps,” explains Favreau. “This is exciting because it suggests that there may be common molecular ‘themes’ associated with cooperation across species.”

A number of the functional groups found to be associated with sociality in this study have also been linked to sociality in other social bees and ants. These include genes related to chromatin binding, DNA binding, regulation of telomere length, and reproduction and metabolism. On the other hand, the study also identified many lineage-specific genes and functional groups associated with social phenotypes. According to the authors, these findings “reveal how taxon-specific molecular mechanisms complement a core toolkit of molecular processes in sculpting traits related to the evolution of eusociality.”

Interestingly, Favreau notes that “a machine learning approach to these large datasets was the best method for uncovering these similarities.” Although the authors first attempted traditional methods for studying differential gene expression, these largely grouped species by phylogeny and failed to identify gene sets associated with sociality. In contrast, machine learning tools provided “a more nuanced and sensitive approach,” allowing the authors to identify gene expression similarities across a wide evolutionary distance.

One remaining question is how the findings of this study, which focused on species with rudimentary forms of sociality, might compare to an obligately eusocial species with morphologically distinct castes of reproductive and nonreproductive individuals. According to Favreau, “This is something we are currently working on and hope to be able to address in the near future. We are taking a broader approach to examine how genes and genomes change during the course of social evolution.” This includes adding transcriptomic data for 16 additional bee and wasp species, enabling “a larger comparative study with species of wasps and bees that are solitary, have rudimentary sociality, and have complex sociality.”

Expansion of the study, however, requires obtaining samples from around the globe, a feat that has at times proved difficult. “It was actually a challenge to find many of these species, some of which had never been studied before on a genetic level!” notes Favreau. “Given the global diversity of taxa and the remote locations many were collected in, we are happy to have been able to obtain all specimens and genomes given the global pandemic and travel restrictions the past few years.” The team was ultimately able to acquire a number of samples through partnerships with other investigators and institutions, emphasizing the critical role of collaboration in scientific discovery.
